# Supplementary material for: Sulforaphane Ameliorates High-Fat-Diet-Induced Metabolic Abnormalities in Young and Middle-Aged Obese Male Mice
Source: Foods. 2024 Mar 29;13(7):1055. doi: 10.3390/foods13071055 (PMC11012181; doi:10.3390/foods13071055)
Supplement: Supplementary file 1 [file foods-13-01055-s001.zip › foods-2925276-supplementary.pptx]

## Slide 1
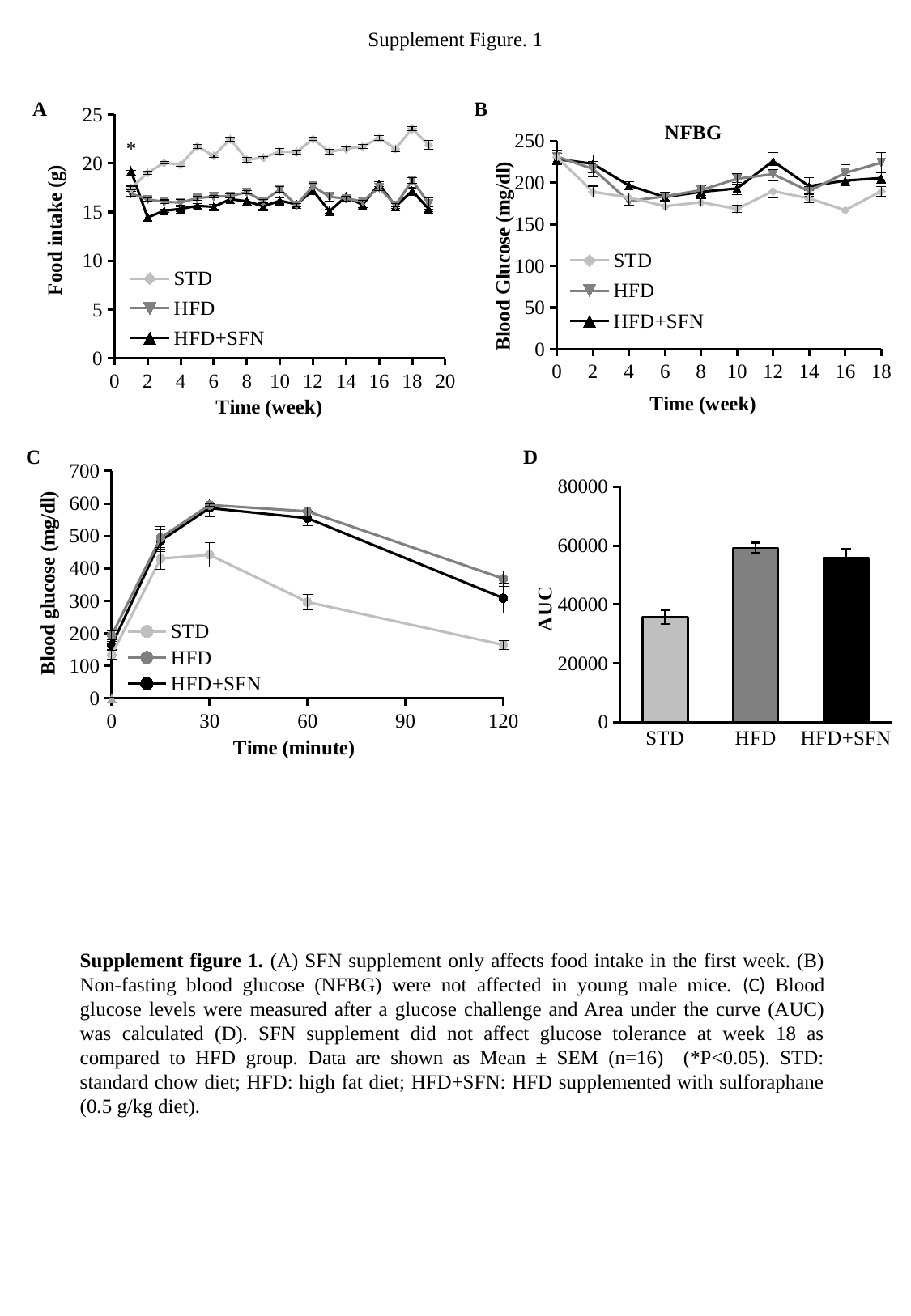

Supplement Figure. 1
B
A
### Chart: NFBG
| Category | STD | HFD | HFD+SFN |
|---|---|---|---|
### Chart
| Category | STD | HFD | HFD+SFN |
|---|---|---|---|*
C
D
### Chart
| Category | STD | HFD | #REF! | HFD+SFN | |
|---|---|---|---|---|---|
### Chart
| Category | |
|---|---|
| STD | 35676.5625 |
| HFD | 59226.5625 |
| HFD+SFN | 55905.9375 |Supplement figure 1. (A) SFN supplement only affects food intake in the first week. (B) Non-fasting blood glucose (NFBG) were not affected in young male mice. (C) Blood glucose levels were measured after a glucose challenge and Area under the curve (AUC) was calculated (D). SFN supplement did not affect glucose tolerance at week 18 as compared to HFD group. Data are shown as Mean ± SEM (n=16) (*P<0.05). STD: standard chow diet; HFD: high fat diet; HFD+SFN: HFD supplemented with sulforaphane (0.5 g/kg diet).

## Slide 2
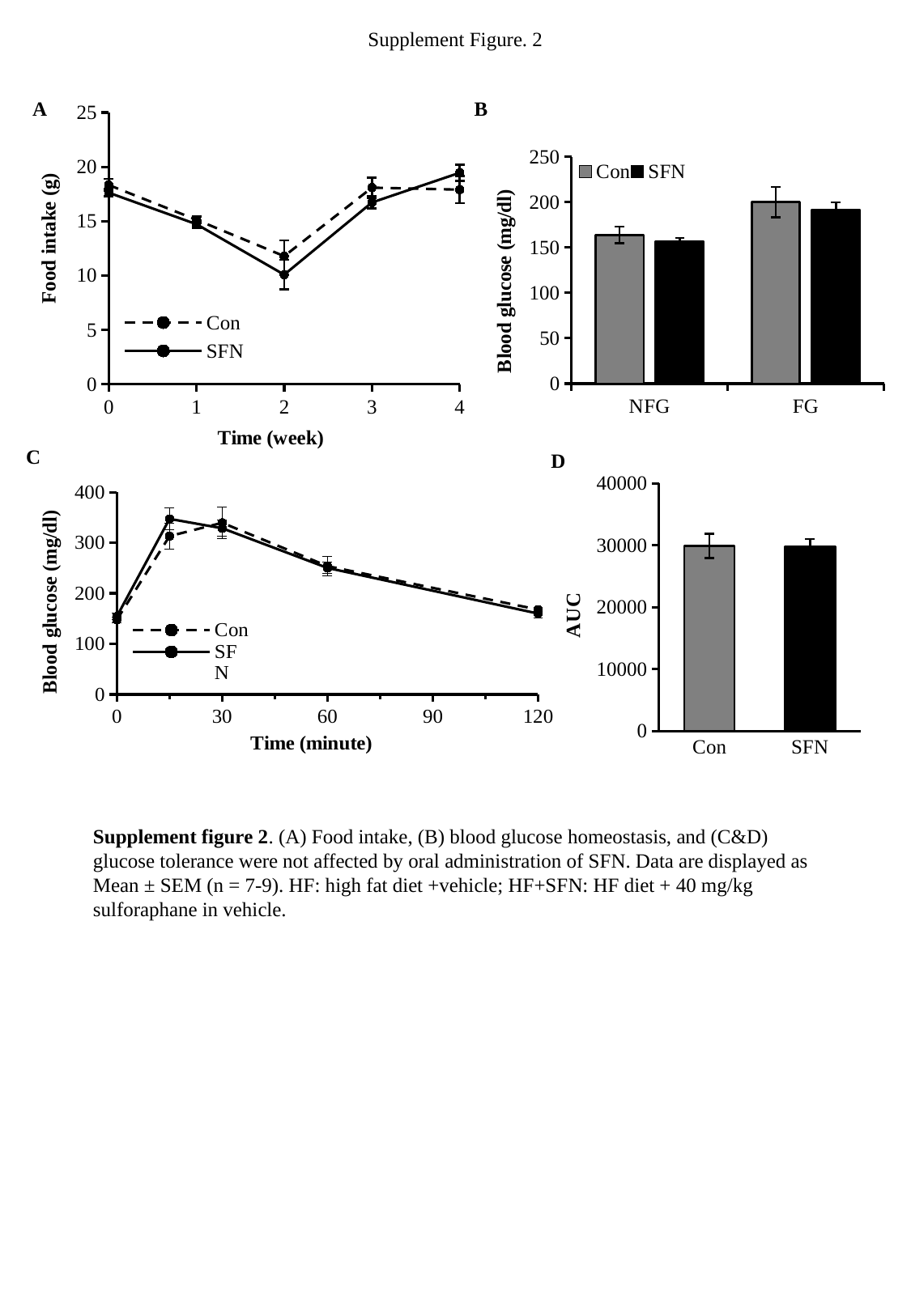

Supplement Figure. 2
B
A
### Chart
| Category | Con | SFN |
|---|---|---|
### Chart
| Category | Con | SFN |
|---|---|---|
| NFG | 163.71428571428572 | 156.88888888888889 |
| FG | 199.85714285714286 | 191.44444444444446 |C
D
### Chart
| Category | |
|---|---|
| Con | 29895.0 |
| SFN | 29817.5 |
### Chart
| Category | Con | SFN |
|---|---|---|Supplement figure 2. (A) Food intake, (B) blood glucose homeostasis, and (C&D) glucose tolerance were not affected by oral administration of SFN. Data are displayed as Mean ± SEM (n = 7-9). HF: high fat diet +vehicle; HF+SFN: HF diet + 40 mg/kg sulforaphane in vehicle.
